# Laparoscopic Management of a Rare Lesser Sac Internal Hernia Involving the Terminal Ileum and Entire Right Colon: A Diagnostic and Surgical Challenge

**DOI:** 10.7759/cureus.87658

**Published:** 2025-07-10

**Authors:** Farhan Akram, Abdulrahman Mohammed, Khurram Siddique

**Affiliations:** 1 General and Colorectal Surgery, The Royal Oldham Hospital, Northern Care Alliance NHS Foundation Trust, Oldham, GBR

**Keywords:** bowel obstruction, foramen of winslow, internal hernia, laparoscopic management, lesser sac

## Abstract

Internal hernias are an uncommon cause of bowel obstruction. The diagnosis and identification of the cause of internal herniation can be challenging, but modern imaging, particularly CT scan, facilitates preoperative identification. The foramen of Winslow hernia represents a rare subtype, accounting for a small fraction of all internal hernias and often presenting with non-specific symptoms. Most reported cases in the literature were managed through open surgery, albeit a few favored laparoscopy. This report presents a challenging case involving herniation of the terminal ileum, cecum, and ascending and transverse colon through the foramen of Winslow. We share the successful laparoscopic management, highlighting the surgical challenges and techniques used in this rare presentation.

## Introduction

Internal hernias are defined as the protrusion of abdominal viscera through defects in the peritoneum or mesentery [1]. They represent less than 1% of all cases of bowel obstruction [2], with an increased incidence noted after bariatric surgical procedures [1,3]. Herniation through the foramen of Winslow is particularly rare, representing about 8% of all internal hernias [1,2,4-6] and 0.08% of all hernias [4,5]. The contents of these hernias are predominantly the small intestine, but the cecum, ascending colon, and other mobile intra-abdominal organs have also been implicated [1,2,4,5,7]. Initially identified by Blandin in 1834 [2], the foramen of Winslow hernia remains an uncommon condition. As reported by Moris et al. [4], approximately 150 cases have been documented in the literature, with only around 15 cases being managed through laparoscopic techniques.

We report a rare case of major herniation through the foramen of Winslow, including the terminal ileum, cecum, and ascending and transverse colon. To the best of our knowledge, this represents the first reported case in the United Kingdom involving herniation of both small and large bowel through the foramen of Winslow. This conclusion is supported by the comprehensive literature review by Moris et al. [4], which identified approximately 150 cases worldwide up to that date, with no reported cases in the United Kingdom involving herniation of both the small and large intestines. A successful laparoscopic repair was performed, and the patient experienced an uneventful postoperative recovery, with complete resolution of symptoms.

## Case presentation

**Figure 1:**
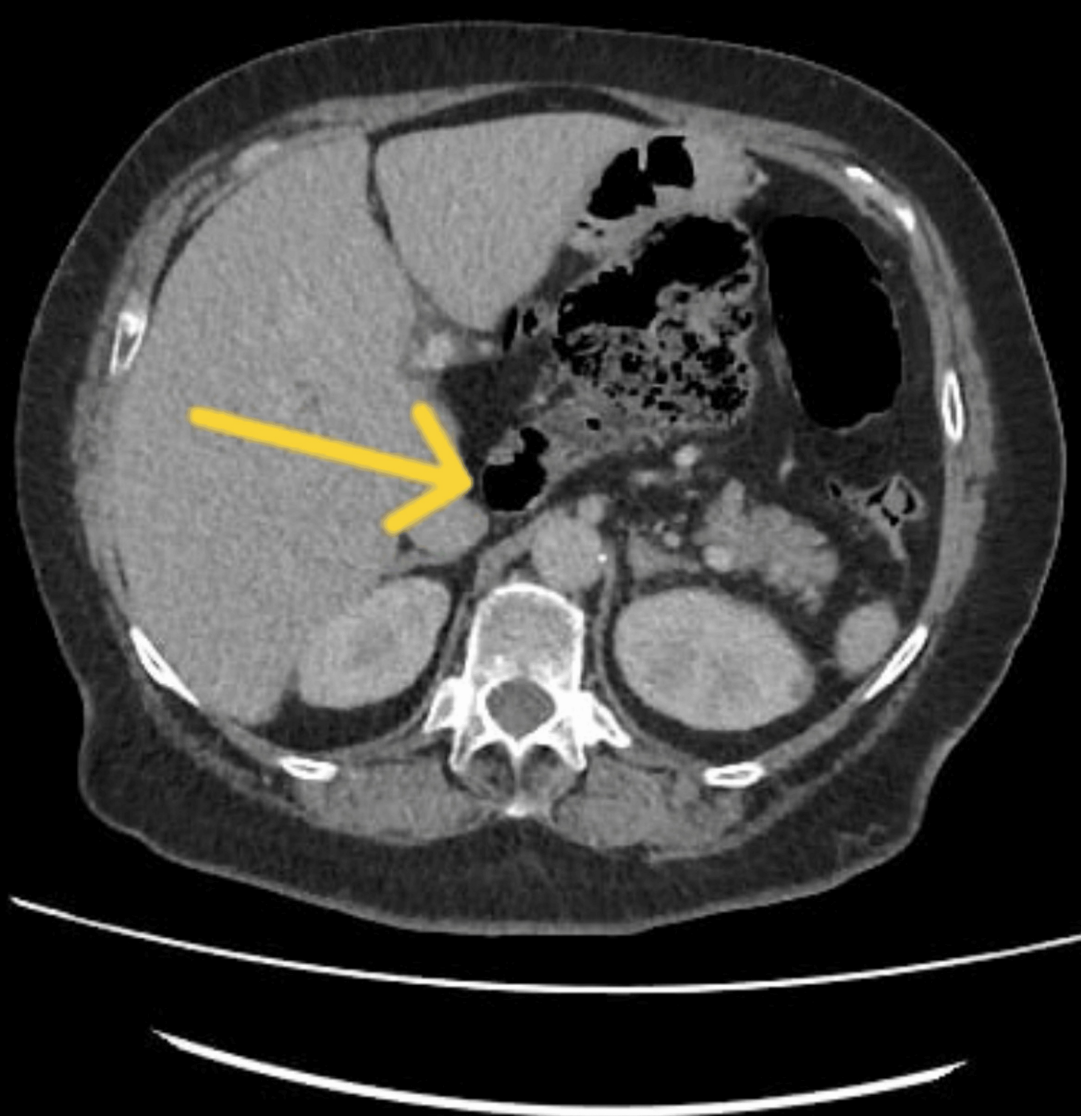
Axial section through the upper abdomen The yellow arrow shows the displaced cecum within the gastrohepatic space at the lesser sac.

**Figure 2:**
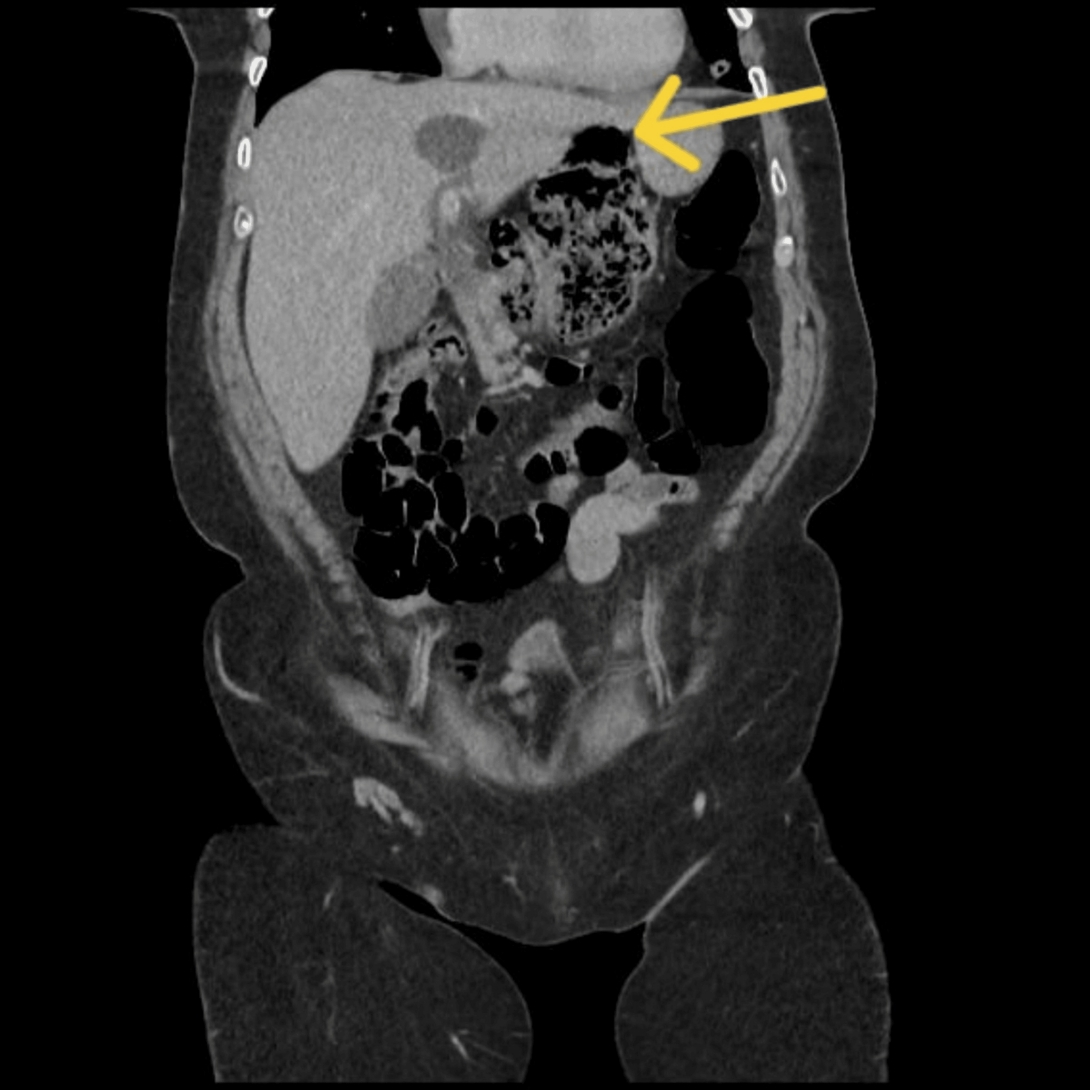
Coronal section The yellow arrow shows part of the colon within the lesser sac.

**Figure 3:**
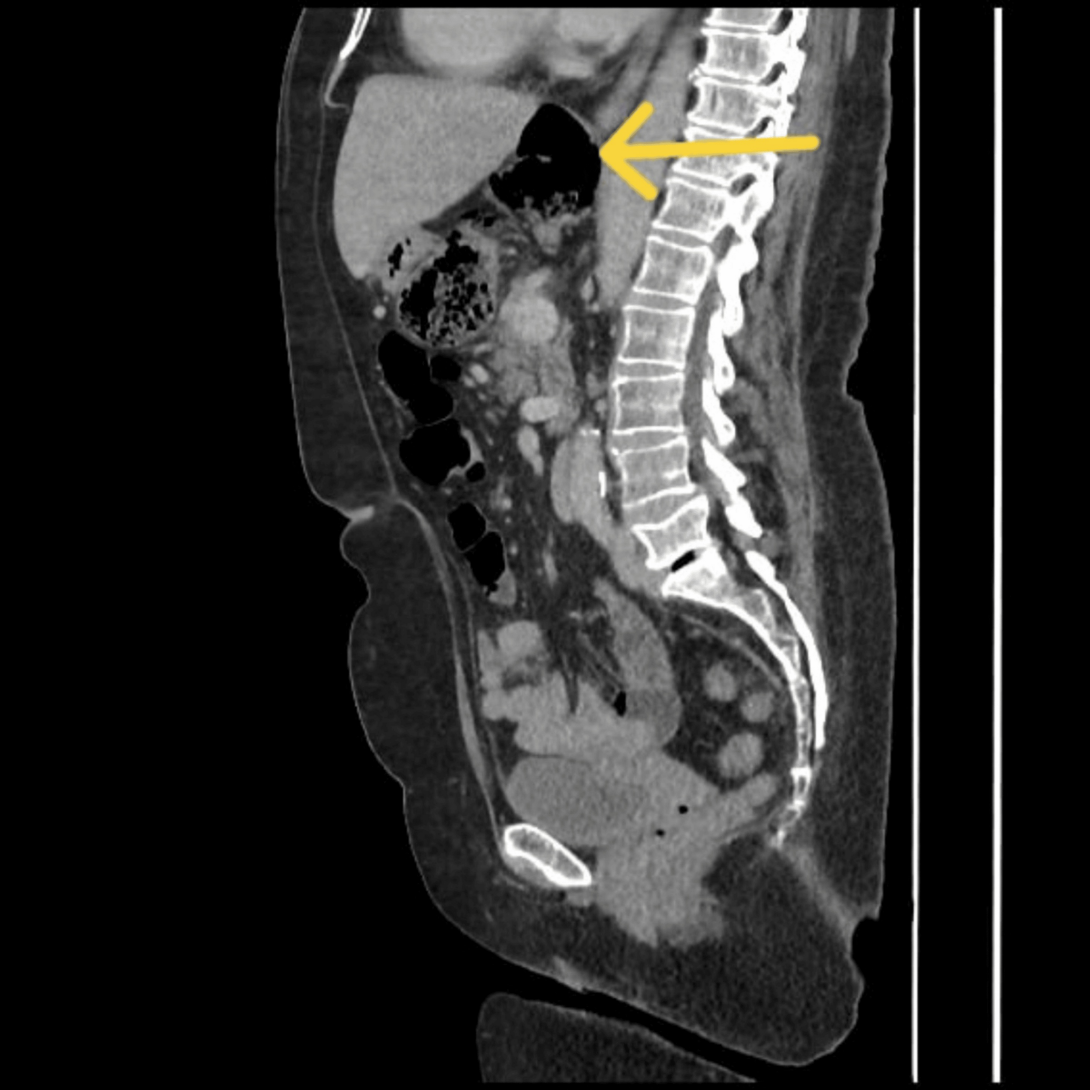
Sagittal section The yellow arrow shows herniation of the right colon and distended cecum within the lesser sac.

A 67-year-old female patient with a history of type 2 diabetes mellitus, chronic obstructive pulmonary disease, hypertension, asthma, and irritable bowel syndrome presented with an acute onset of severe upper abdominal pain for two days, along with nausea and vomiting. She had no prior history of similar abdominal symptoms or hospital admissions. Her clinical examination was unremarkable, with no signs of peritonitis. Her inflammatory markers were normal. Contrast-enhanced CT of the abdomen and pelvis identified a moderate-sized lesser sac hernia involving the cecum and right colon, causing mass effect, with features of subacute bowel obstruction and flattening of the portal vein (Figures 1-3).

**Figure 4:**
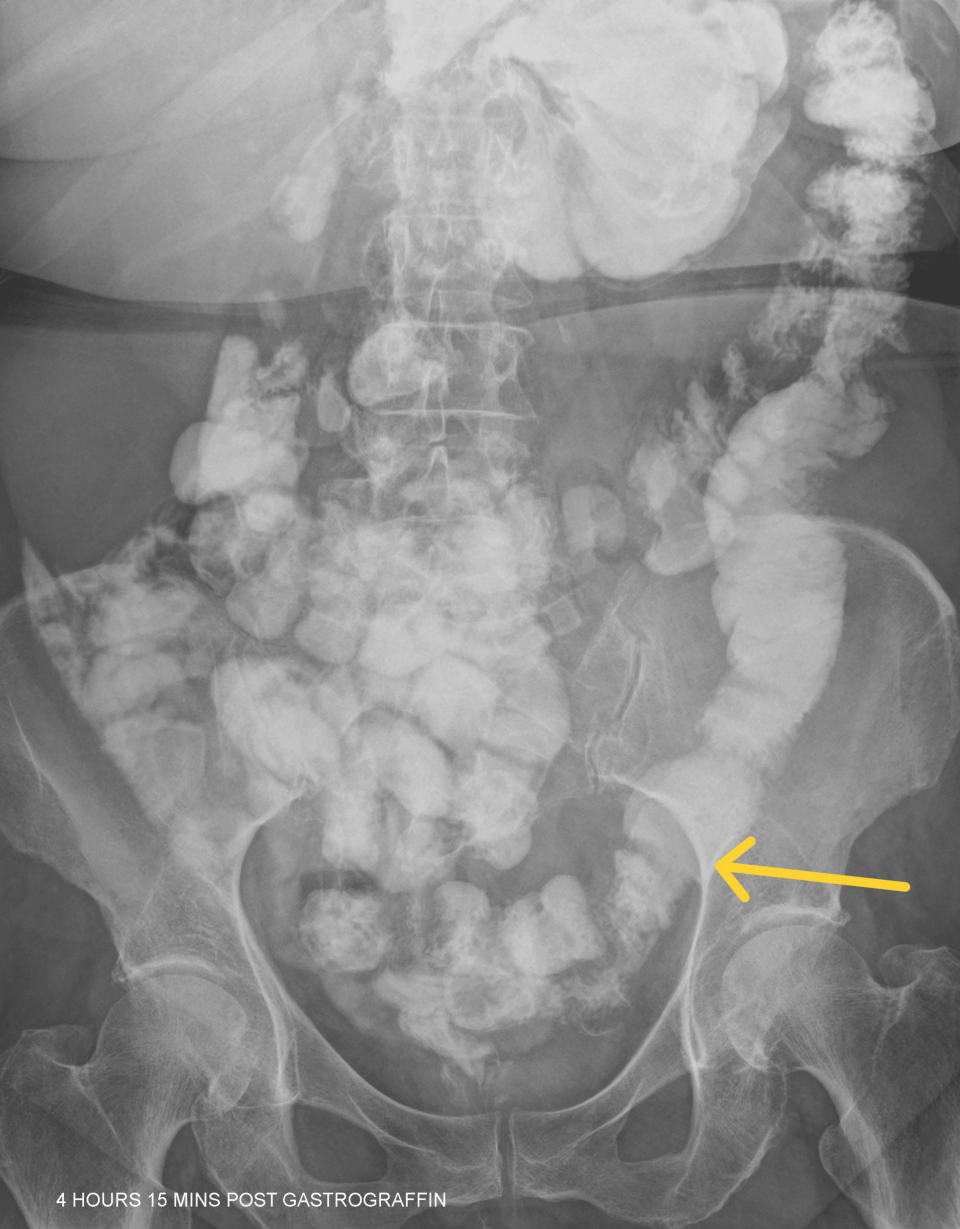
Abdominal X-ray after Gastrografin The yellow arrow demonstrates that the Gastrografin has successfully reached the sigmoid colon.

The patient was initially managed conservatively and underwent a Gastrografin study to confirm resolution of mechanical obstruction. Following the administration of Gastrografin, she successfully passed a bowel movement. An abdominal X-ray confirmed that the distal small bowel loops were of average calibre, thereby excluding the possibility of a high-grade obstruction. Furthermore, the oral contrast medium had advanced to the sigmoid colon, as illustrated in Figure 4, with opacification of the remaining colon. Given the resolution of symptoms and reassuring radiological findings, the patient was considered suitable for discharge with scheduled outpatient follow up.

The patient was readmitted within 48 hours due to recurrence and worsening symptoms, necessitating diagnostic laparoscopy. Laparoscopic exploration revealed a complex internal herniation of the terminal ileum, ascending colon, cecum, and proximal transverse colon through a narrowed foramen of Winslow. Findings included dense omental adhesions and anatomical distortion. The hepatoduodenal ligament remained intact, and the free edge of the foramen of Winslow was divided carefully, protecting the hepatoduodenal ligament contents. The bulky herniated bowel was reduced and was viable with visible peristalsis. Due to the abnormal mobility of the cecum and the risk of volvulus, a cecopexy was performed, and the mildly inflamed appendix was removed. The defect was then repaired. Operative time was approximately 90-100 minutes, with minimal blood loss. The laparoscopic video demonstrates a step-by-step approach to safely repair the challenging hernia (Video 1).

**Video 1:** Laparoscopic repair of a foramen of Winslow hernia containing the terminal ileum, cecum, ascending colon, and transverse colon, followed by cecopexy

Postoperative recovery was uneventful, and the patient was discharged on the third day. One month postoperatively, the patient remained clinically well on follow-up, with no new concerns reported.

## Discussion

The herniation through the foramen of Winslow, also known as the epiploic foramen, is an infrequent form of internal abdominal hernia, accounting for fewer than 1% of all reported cases of internal hernias [4]. This normal anatomical orifice is bounded by the caudate lobe of the liver, duodenum, inferior vena cava, and hepatoduodenal ligament [4,5]. Most commonly, the small bowel herniates through the foramen of Winslow, followed by the ascending colon (30%) and transverse colon (7%) [4,6]. In our case, the involvement of the terminal ileum, cecum, and ascending and transverse colon is notable and rarely reported.

Diagnostic challenges and preoperative imaging

Clinical presentation is usually non-specific, with intermittent abdominal pain, distension, or signs of obstruction, often delaying diagnosis [5,6]. Literature indicated that nearly 90% of foramen of Winslow hernias are discovered intraoperatively. However, the increased use of CT has improved preoperative diagnosis [8]. A review from 2018 to 2023 identified 21 reported cases, with only three cases (14%) diagnosed intraoperatively [7]. The CT scan of the abdomen and pelvis in our patient delineated the internal herniation through the foramen of Winslow; however, it did not clearly identify the volume or specific contents of the herniated viscera.

Laparoscopic vs. open surgical repair

Open surgery was favored traditionally because of the technical difficulties involved in the reduction of herniated contents and assessing the viability of the bowel. However, our case demonstrates the feasibility of laparoscopic management, even in complex scenarios, with benefits including: (i) reduced postoperative morbidity, (ii) enhanced anatomical visualization, and (iii) safe reduction of multiorgan herniation with adjunctive procedures such as cecopexy and appendicectomy [4,8]. Laparoscopy requires meticulous adhesiolysis and careful handling of the bowel to avoid serosal injury [8,9].

Most reported cases involve only the small bowel [1,4,5]. Involvement of both small and large bowel, as seen in our case, is rare and may be attributed to a wider foramen or congenital ligamentous laxity [10]. According to a literature review by Honma et al. [11], only six such cases have been documented. Five of these cases involved the terminal ileum and cecum, and one involved the terminal ileum and right colon. This anatomical variant necessitates: (i) intraoperative viability assessment and (ii) consideration of prophylactic fixation (cecopexy) to prevent recurrence [10]. To the best of our knowledge, this is the first reported case in the United Kingdom of combined small and large bowel herniation through the foramen of Winslow.

Laparoscopic management of foramen of Winslow hernia has proven to be a safe and efficient alternative to laparotomy, with good outcomes reported and no cases of recurrence in the literature. Our patient benefited from the laparoscopic approach and had a short length of stay with no complications. On follow-up evaluation, she remained asymptomatic with complete clinical resolution.

## Conclusions

Foramen of Winslow hernias, though rare, carry a high risk of mortality unless identified and treated promptly. While rarely reported, laparoscopic repair is a valid and effective option in selected patients. This case emphasizes the need for clinical acuity, the timeliness of imaging, and the effectiveness of a minimally invasive technique in this complicated setting.
